# Supplementary material for: The Dual Prey-Inactivation Strategy of Spiders—In-Depth Venomic Analysis of Cupiennius salei
Source: Toxins (Basel). 2019 Mar 19;11(3):167. doi: 10.3390/toxins11030167 (PMC6468893; doi:10.3390/toxins11030167)
Supplement: Supplementary file 1 [file toxins-11-00167-s001.zip › Supplementary Fig S7/Supplementary Fig S7.pdf]

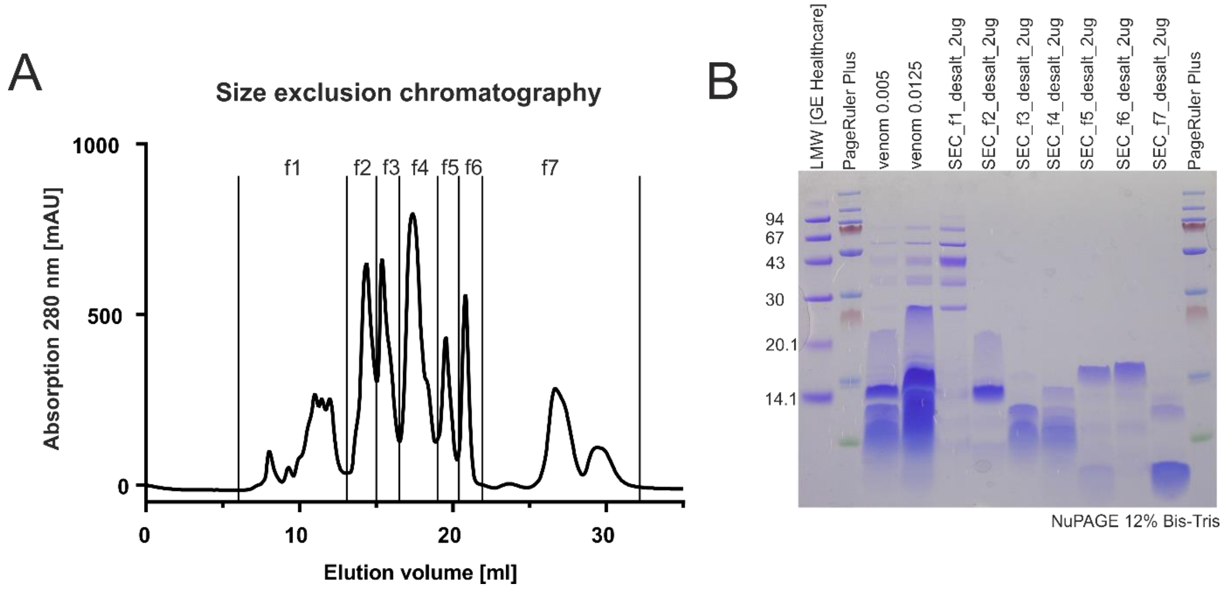

Supplementary Fig S7. Separation of *C. salei* venom for proteomics.

A. Size exclusion chromatography of 50 µl *C. salei* venom on a Superdex 75 Increase HR 10 / 300 column (GE Healthcare) using 50 mM NH4OAc pH 5.0 supplemented with 500 mM NaCl as liquid phase. The seven venom fractions collected for further proteomic analysis are indicated by vertical lines.
B. SDS-PAGE of collected fractions on a NuPAGE 12% Bis-Tris gel (Invitrogen).
